# Diclofenac induced anaphylaxis: A case report from Nepal

**DOI:** 10.1016/j.amsu.2022.104233

**Published:** 2022-07-31

**Authors:** Himal Bikram Bhattarai, Sangam Shah, Manish Uprety, Santosh Baniya, Gyabina Maharjan, Anshu Priya, Prabesh Bikram Singh, Ayusha Subedi

**Affiliations:** a Gandaki Medical College and Teaching Hospital, Pokhara, Nepal; b Tribhuvan University, Institute of Medicine, Maharajgunj, 44600, Nepal; c Kathmandu University School of Medical Sciences, Dhulikhel, Nepal; d Metrocity Hospital, Pokhara, Nepal; e Department of Internal Medicine, Kirtipur Hospital, Kathmandu, Nepal; f Janaki Medical College, Janakpur, Nepal; g All Nepal Hospital, Samakhusi, Nepal; h Manmohan Memorial Community Hospital, Jhapa, Nepal

**Keywords:** Diclofenac, Anaphylaxis, Intravenous

## Abstract

**Introduction:**

Diclofenac is considered a generally safe medication, and cause few side effects like dyspepsia, diarrhea/constipation, nausea, and vomiting, stomach bleeding, rash, urticaria, photosensitivity reactions, acute renal failure, analgesic nephropathy, bone marrow and liver diseases. Rarely, it causes anaphylactic reaction.

**Case presentation:**

We report a case of 40 years male who developed anaphylactic reaction after intravenous infusion with diclofenac.

**Discussion:**

Both the pulmonary embolism and anaphylaxis guidelines emphasize the importance of a thorough evaluation when making a diagnosis. Mast cells secrete tryptase, a neutral protease that is specifically concentrated in the secretory granules of human mast cells (but not basophils), along with histamine, as a marker of mast cell activation.

**Conclusion:**

Although intravenous diclofenac sodium is a safe and often used medicine, it can cause severe and perhaps fatal anaphylactic responses.

## Highlights

• IV diclofenac sodium can cause fatal anaphylactic responses.
• It might mimic pulmonary embolism in symptoms.
• Accurate and timely diagnosis of anaphylaxis and withheld of diclofenac should be done.

## 1 Introduction

Nonsteroidal anti-inflammatory medications (NSAIDs) include a variety of agents with different chemical structures. Anti-inflammatory (inflammatory response modulation), analgesic (pain alleviation), and antipyretic (antipyretic effects) are the three main activities of these medications (fever reduction). All of this is tied to NSAIDs' principal impact, which is to limit prostaglandin and thromboxane production by reducing cyclooxygenase. Arachidonic acid metabolism involves the cyclo-oxygenase and lipo-oxygenase pathways. Both pathways create powerful mediators of immunological and inflammatory responses. Any blockage of the cyclo-oxygenase pathway (COP) redirects metabolism to the lipo-oxygenase pathway (LOP), which may increase the system's negative effects by boosting cysteinyl-leukotriene synthesis and release [1,2].

Side effects include dyspepsia, diarrhea/constipation, nausea, and vomiting, as well as stomach bleeding and ulcers, rash, urticaria, photosensitivity reactions, acute renal failure, analgesic nephropathy (with long-term use), and bone marrow and liver damage [3]. Diclofenac is a nonopioid analgesic that is widely used around the world. Only a few anaphylactic responses have been observed, making it a usually safe drug. Anaphylactic reaction to diclofenac, given as an intravenous infusion, is described. This case was reported in accordance with SCARE 2020 guidelines [4].

## 2 Case presentation

A 40 years old male presented to the emergency with sudden onset of shortness of breath with pleuritic chest pain, and rashes which extended all over the body within minutes. He had history of abdominal discomfort and pain for one week. The pain increased in one day for which he was given intravenous diclofenac in local clinic 10 minutes prior to presentation at our center. He has no known allergy to drug or any other substances. He underwent polypectomy 2 month back for nasal polyp.

At the time of presentation, he was on severe respiratory distress with oxygen saturation 67% at room temperature, respiratory rate of 30/min, blood pressure 80/60 mmHg, pulse rate was 150 beats per minute, low in volume, regular, and normal in character, and body temperature of 37.7 ֩C. On auscultation of chest, he had diffused wheeze bilaterally. The serum tryptase level was increased and methylhistamine was found in his urine. Laboratory investigations revealed elevated D-dimer (3.6 mg/l) (Ref range:<250 ng/ml) and serum tryptase (25 ng/ml) (Ref range: 3–5 ng/ml). Methylhistamine level in the urinary sample taken about 4 hours later was 136 ng/μmol. Other laboratory investigations were within normal range.

Patient was stabilized with epinephrine, antihistamines, intravenous fluid, and oxygen therapy via facemask along with hydrocortisone 200mg three times a day (TDS). He was hospitalized and kept in intensive care unit (ICU) for 24 hours. He didn't develop biphasic or multiphasic anaphylactic episodic complications during the hospital stay. Following the recovery, the patient was discharged on antihistamine for one week. On follow-up after a week, he was improving and there were no fresh issues.

## 3 Discussion

NSAID hypersensitivity can present itself in a variety of ways, from urticaria to life-threatening anaphylaxis. Patients with a history of allergic reactions or autoimmune illnesses are more likely to experience these reactions, making them a high-risk population [2]. Diclofenac was once thought to be a safe medicine, with a lot of patient-years under its belt. Other nonsteroidal anti-inflammatory drugs (NSAIDs) have comparable negative effects. There have been several reports of severe anaphylactic responses after taking diclofenac, some of which were deadly [5]. The case studies demonstrate that anaphylaxis can occur regardless of the route of administration whether the diclofenac sodium is administered intramuscularly, orally, rectally, or intravenously [[5], [6], [7]]. Anaphylactic reactions have been documented to produce cardiovascular events such as myocardial infarction and acute coronary syndromes in patients with healthy coronary arteries. Transfusion-related acute lung injury (hypoxia, bilateral chest infiltrates) and sudden acute reactionary hemorrhage (tachycardia, hypotension, but soft abdomen, no pallor) were also considered as differential diagnoses, but were ruled out later.

When it comes to diagnosing pulmonary embolism and anaphylaxis, both guidelines emphasize the significance of a comprehensive examination. As a sign of mast cell activation, mast cells secrete tryptase, a neutral protease that is uniquely concentrated in the secretory granules of human mast cells (but not basophils) [8,9]. Another sign is urinary methylhistamine, a histamine metabolite with a longer half-life (2–3 hours) than histamine [10]. These markers can be utilized to offer a retrospective diagnosis after the patient has completed their initial therapy. The levels of tryptase in our patient's blood were elevated, and methylhistamine was discovered in his urine, indicating an allergic reaction.

The D-dimer test, when used in conjunction with a clinical likelihood assessment, can assist patients rule out pulmonary embolism. It has little specificity and is elevated in a variety of diseases, including anaphylaxis, due to allergic processes initiating the coagulation cascade [11]. High levels of D-dimer were found in this case, which is unsurprising. The pulmonary infiltrates found in our patient were most likely caused by diclofenac therapy, as previously observed [12]. The Naranjo likelihood scale suggested that diclofenac was likely to be the cause of the occurrence (score 8) [13].

A thorough clinical evaluation is required in order to begin proper treatment as soon as possible in order to avoid future morbidity and mortality. Epinephrine is the first and most important treatment for anaphylaxis, and it should be taken whenever possible because to its relative safety [14]. When clinical signs suggest acute pulmonary embolism in a patient, anticoagulation can be begun empirically, as there is a correlation between early anticoagulation and lower mortality in patients with acute pulmonary embolism [15].

As an immunological disorder, hypersensitivity is characterized by excessive or inappropriate immune response, which is frequently directed towards harmless antigens and results in tissue damage. Type I (immediate), type II (antibody-mediated), type III (immune complex-mediated), and type IV (cell-mediated or delayed-type) hypersensitivity are the four categories under which hypersensitivity can be divided. The most prevalent immunological condition, type I hypersensitivity or allergy, is mostly mediated by mast cells and immunoglobulin (Ig)E. By three basic mechanisms—direct cellular death, inflammation, and disruption of cellular function—type II hypersensitivity can cause tissue injury. Type III hypersensitivity is brought on by either excessive immune complex formation or poor clearance. T lymphocytes and macrophages are the mediators of type IV hypersensitivity. Our case is type I hypersensitivity reaction induced by anaphylaxis.

## 4 Conclusion

This is the first case of anaphylactic response caused by IV diclofenac that we are aware of from Nepal. This campaign tries to increase awareness that, while IV diclofenac sodium is a common and safe medication, it can cause severe and perhaps fatal anaphylactic reactions. Because the symptoms may resemble those of pulmonary embolism, doctors should be aware of this dangerous, albeit uncommon, side effect of IV diclofenac to ensure an accurate and quick diagnosis. We recommend usual testing against diclofenac.

## Provenance and peer review

Not commissioned, externally peer-reviewed.

## Consent

Written informed consent was obtained from the patient for publication of this case report and accompanying images. A copy of the written consent is available for review by the Editor-in-Chief of this journal on request.

## Registration of research studies

1. Name of the registry: None
2. Unique Identifying number or registration ID: None
3. Hyperlink to your specific registration (must be publicly accessible and will be checked):

## Guarantor

Dr. Himal Bikram Bhattarai.

## Ethical approval

None.

## Sources of funding

No funding was received for the study.

## Author contribution

SS and HBB wrote the original manuscript, reviewed, and edited the original manuscript. MU, SB, GM, AP, AS, and PBS reviewed and edited the original manuscript.

## Declaration of competing interest

Authors have no conflict of interest to declare.
